# Cryptic Fitness Advantage: Diploids Invade Haploid Populations Despite Lacking Any Apparent Advantage as Measured by Standard Fitness Assays

**DOI:** 10.1371/journal.pone.0026599

**Published:** 2011-12-09

**Authors:** Aleeza C. Gerstein, Sarah P. Otto

**Affiliations:** Zoology Department, Biodiversity Research Center, The University of British Columbia, Vancouver, British Columbia, Canada; University of Minnesota, United States of America

## Abstract

Ploidy varies tremendously within and between species, yet the factors that influence when or why ploidy variants are adaptive remains poorly understood. Our previous work found that diploid individuals repeatedly arose within ten replicate haploid populations of *Saccharomyces cerevisiae*, and in each case we witnessed diploid takeover within 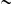
1800 asexual generations of batch culture evolution in the lab. The character that allowed diploids to rise in frequency within haploid populations remains unknown. Here we present a number of experiments conducted with the goal to determine what this trait (or traits) might have been. Experiments were conducted both by sampling a small number of colonies from the stocks frozen every two weeks (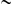
93 generations) during the original experiment, as well through sampling a larger number of colonies at the two time points where polymorphism for ploidy was most prevalent. Surprisingly, none of our fitness component measures (lag phase, growth rate, biomass production) indicated an advantage to diploidy. Similarly, competition assays against a common competitor and direct competition between haploid and diploid colonies isolated from the same time point failed to indicate a diploid advantage. Furthermore, we uncovered a tremendous amount of trait variation among colonies of the same ploidy level. Only late-appearing diploids showed a competitive advantage over haploids, indicating that the fitness advantage that allowed eventual takeover was not diploidy per se but an attribute of a subset of diploid lineages. Nevertheless, the initial rise in diploids to intermediate frequency cannot be explained by any of the fitness measures used; we suggest that the resolution to this mystery is negative frequency-dependent selection, which is ignored in the standard fitness measures used.

## Introduction

The study of adaptive evolution is in many ways the study of fitness. Having identified an interesting pattern in nature, we examine and compare fitness differences within contemporary populations to infer how evolution might have happened. This method has notoriously been criticized by one of the most widely cited papers in the field [1] because we traditionally lack the ability to perform direct experiments on the individuals that were actually present at the time when evolution occurred to determine which mutation provided an advantage (and why). Recently, experimental evolution with microbes has provided an approach whereby the entire process of evolutionary change can be studied and used to test adaptive processes directly, without inference about the populations and individuals involved.

Experimental evolution allows researchers to conduct experiments forward in time. By maintaining freezer stock of samples taken from the population at multiple timepoints during evolution (generating a “fossil record”), researchers are able to ask questions about when an adaptation first arose, how rapidly it rose in frequency, and what enabled it to predominate over previous genotypes. Experimental evolution studies are typically initiated by starting many replicate populations of the same (ancestral) genotype; thus this approach has been a fruitful way to explore the range of paths that evolution can take, given the same starting material.

Our previous work reported a surprising result that arose during an 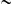
1800 generation batch culture evolution experiment. We found that diploid individuals arose within haploid populations of *Saccharomyces cerevisiae* and eventually swept independently in ten lines, even though the lines were asexual (5/5 lines evolved in YPD & 5/5 lines evolved in YPD+salt, [2]). We proposed that historical contingency may be acting; as *S. cerevisiae* is historically diploid, selection may have acted on rare diploid individuals that arise naturally at low frequency to regain this historical state. However, the true character on which selection was acting to allow diploids to take over remains unknown. Here, we present experiments conducted with the goal of determining what fitness component allowed diploids to repeatedly invade haploid populations.

The question of why one ploidy level is able to outperform another over evolutionary time is of broad interest, as tremendous variation in ploidy is seen throughout the tree of life, even among closely related species [3]. All sexual species undergo a ploidy cycle over a generation, and some species maintain prominent haploid and diploid free-living stages (i.e., alternation of generations), while in other species ploidy differs between sexes (i.e., haplodiploidy, e.g., in Hymenoptera). Though ploidy variation is pervasive, we generally have a poor understanding of when or why ploidy variants are adaptive. As one example, a recent study that examined the link between ploidy and plant species worldwide found endangered plants were disproportionately diploid, while invasive species were more likely to be polyploid [4], yet the traits that underlie these correlations remain speculative.

The species we focus on, *Saccharomcyes cerevisiae*, is itself known to display multiple ploidy levels in natural isolates [5]. Ploidy variants of *S. cerevisiae* are known to differ in a wide variety of aspects, even when isogenic. Cell size increases as ploidy increases [6], [7]; as *S. cerevisiae* cells are prolate spheroids, increases in volume decreases the surface area to volume ratio, and thus diploids, which are typically larger than haploids, have a significantly lower surface area to volume ratio. Gene expression and protein levels also differ between isogenic individuals; deGodoy *et al*. [8] found that 196 proteins changed more than 50% in abundance between haploids and diploids, while Wu *et al*. [9] recently observed that 65 genes differ in expression between haploid and tetraploid *S. cerevisiae* isogenic individuals. Interestingly, it may be cell size rather than ploidy that influences gene expression, as Wu *et al*. also showed that genes with expression differences between haploids and tetraploids also differed in expression (in the same direction) when comparing wild type haploids with *cln3* 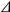 haploids that are 185% the volume of wildtype haploids.

Previous researchers have found mixed results when comparing haploid and diploid fitness under conditions similar to ours (isogenic haploids and diploids grown in rich medium at 30

C). Adams & Hansche [10] and Temina *et al*. [11] found no difference in growth rate between haploids and diploids under these conditions, while Mable [7] found haploids grew significantly faster than diploids (though this was not significant after correcting for multiple comparisons). Similarly, although Glazunov *et al*. [12] found diploids outcompeted haploids, Mable [7] found haploids and diploids competed equally well against a common haploid or diploid competitor in YPD at 30

C. Overall, previous work in our lab and others has failed to identify any distinct fitness advantage of diploids over haploids under the conditions of our long-term experiment.

Here we set out specifically to determine why diploid individuals, when they arise by chance within haploid populations, were able to out perform haploids under our experimental conditions. To identify the character that might have allowed diploids to overtake haploids, we conducted a set of fitness assays on haploid and diploid individuals isolated at regular intervals throughout the time series of the original experiment (1767 generations). We can thus assess how different fitness components changed throughout the duration of the experiment. We also conducted competitive fitness assays by directly competing haploid and diploid individuals isolated from the same time point. We focus our attention on two time points in particular, where the diploids have recently risen to appreciable frequency (appreciable enough to be sampled), suggesting a recent selective advantage.

We first assayed cell size and shape of haploid and diploid colonies isolated throughout the time series to gain a sense of the magnitude of phenotypic change. We then compared fitness between haploid and diploid genotypes in a variety of ways. It was important to assay many possible aspects of total fitness, as a previous study that acquired mutations through mutation accumulation in *S. cerevisiae* for 1012 generations found that mutations that altered one component of fitness generally had little effect on other components [13]. We thus assayed colonies for three fitness components that correlate to the three main phases of growth during batch culture, i.e., lag time upon entering fresh medium, growth rate during logarithmic growth, and biomass production (yield) after 24 hours of growth (transfer into fresh medium was done every 24 hours in our original experiment). We then conducted two types of competition assays: in the first we compete all individuals of interest against a common competitor (closely related to the ancestor), in the second we developed a novel assay that allowed us to directly compete haploid and diploid genotypes isolated from the same time point against each other. We found, surprisingly, that none of these assays indicate a clear diploid advantage that could explain how diploid genotypes were able to rise in frequency within the initially haploid populations. One possibility is that the initial rise in the frequency of diploidy was due to an aspect of fitness not measured by any of these metrics, and we suggest that frequency-dependence may be involved. We also observed significant variation among colonies of the same ploidy level isolated at the same time point. The eventual fixation of diploidy involved a strain that did exhibit a competitive advantage, suggesting that only a subset of diploids could rise to fixation. We conclude that although the end evolutionary result may be deterministic (i.e., that diploids repeatedly take over the population) the route to takeover appears to be largely stochastic, depending on the exact genotypes that arise.

## Materials and Methods

### Isolating ploidy variants

We previously reported the convergence of 10 replicate haploid lines towards diploidy during 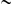
1800 generations of batch culture evolution (1767 generations total) [2]. The ancestral strain haplotype is *MATa-a1* *ste6* 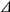 *8-694 ura3* 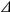 *0 leu2* 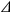 *0 his4* 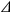 *0 trp1* 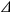 *0 can1* 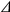 *0*. The mutation at the *MAT* locus and *STE6* partial deletion should ensure complete asexuality; previous work found no evidence of revertants at the *MAT* locus or evidence of sexual reproduction [2]. This past work reported a snapshot of genome size change, by assaying the ploidy level of only a single colony from each of the 10 lines every 93 generations up to generation 744, and a single colony from each line at generation 1767. These colonies were obtained from stocks frozen every two weeks in the original experiment (corresponding to Log
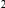
101*14 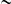 93 generations with daily 1∶101 dilutions in batch culture). Here we focus on only one line (“Line A”) that had been grown in YPD and showed complete diploid takeover by generation 1488 (Figure S1), but analyze multiple colonies from multiple time points to gain a more complete sense of the relative number and fitness of haploid and diploid individuals.

**Figure 1:**
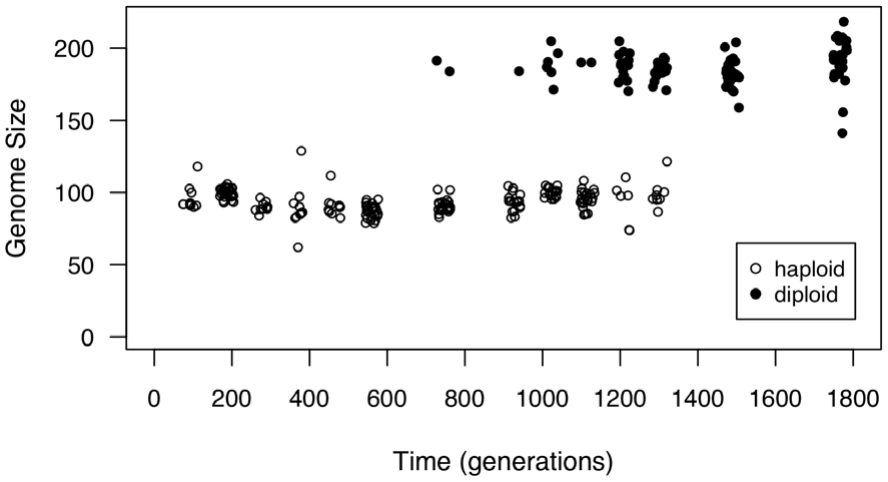
Polymorphism for genome size across the time series. 30 000 cells from each of 24 colonies were measured on a FACSCalibur at each time point, with haploid and diploid assignment determined by the kmeans function in the R programming language [15]. Points are plotted with slight jitter on the x-axis for viewing purposes.

We first isolated ploidy variants from throughout the time series. Freezer stock acquired during the initial evolution experiment [2] was streaked onto YPD plates and allowed to grow for 72 hours. 24 colonies from each time point were haphazardly picked, inoculated into 10 mL YPD and allowed to grow overnight. Flow cytometry on a FACSCalibur was performed as previously described [2] to assay the ploidy of each colony. Culture from these isolated colonies of known ploidy were then frozen in 15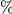 glycerol for use in all later experiments. We found extensive polymorphism for ploidy from generation 744 to generation 1302 (Results, Figure 1), which allowed us to undertake the experiments described below.

We designed a number of experiments to determine whether the ploidy of a sampled colony directly influenced different components of fitness. We first assayed a small number of colonies (two to four) from approximately every 93 generations over the entire 1767 generation time series. These data allowed us to ask whether the fitness measures changed over evolutionary time during the evolution experiment and whether haploid and diploid colonies responded differently. We then took a more in depth look at colonies from generation 1023 and generation 1302. These time points were chosen specifically, as these are the first and last timepoints where polymorphism for genome size was prevalent, and we believed that they would shed the most light on the relative fitness advantage that allowed diploids to invade. We randomly picked five haploid and five diploid colonies (of the 24 initial colonies assayed) from each of these time points and used these same twenty colonies (2 timepoints 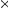 (5 haploid colonies+5 diploid colonies)) for all subsequent assays. We should note that we do not know whether multiple colonies isolated at the same time point are different genotypes, how many times diploid colonies independently arose, or whether colonies isolated at later generations are the direct descendants of colonies isolated earlier.

### Cell size and shape

We first conducted an imaging experiment to measure the cell size and shape of haploid and diploid colonies isolated throughout the time series. As these parameters are known to differ between cells of different ploidy, they may directly contribute to fitness differences, as well as indicate the magnitude to which evolution acted within 1767 generations. We assayed colonies from across the entire time series and from twenty colonies isolated at generations 1023 and 1302. The imaging experiment was initiated by streaking colonies onto plates from freezer stock kept at −80

C and allowed to grow for 72 hours. One colony from each line of interest was then randomly picked, inoculated into 10 mL of YPD and grown shaking at 30

C for 24 hours. One slide was prepared from each culture using standard practices. A Zeiss Axioplan microscope with a digital camera attached was used to take at least three digital pictures of each slide (see Figure S2 for representative haploid and diploid images). Fifteen individual cells were randomly chosen from across the pictures (any cell touching another cell or in the process of budding was excluded). Using the software ImageJ (available at http://rsb.info.nih.gov/ij; developed by Wayne Rasband, National Institutes of Health, Bethesda, MD), photos were enhanced and ellipses were manually drawn around the perimeter of each chosen cell to obtain a length measurement (major axis, L) and width measurement (minor axis, W). We calculated two cell size parameters using the appropriate equations for prolate spheroids, volume (V) and surface area (SA):
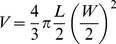
(1)

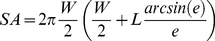
(2)


The equation for surface area depends on the measure of eccentricity (e), which we also used as a descriptor of cell shape:
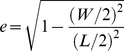
(3)


Lastly, we calculated the surface area to volume ratio (SA/V), which also describes a component of cell shape.

### Fitness components

We first measured various fitness components to test whether there were consistent and significant differences over time and between haploid and diploid colonies. We picked three fitness assays that largely reflect the different phases of *S. cerevisiae* growth in YPD during the 24 hours between transfers in our primary experiment. A brief lag phase occurs after transfer into fresh medium, before cells begin growing, followed by a phase of exponential growth during which *S. cerevisiae* rapidly grow and reproduce by fermenting glucose. A diauxic shift between glucose fermentation and ethanol respiration typically occurs around 20 hours for wildtype cells grown in YPD [14]. During this postdiauxic phase *S. cerevisiae* grows much slower by respiring the ethanol that is a byproduct of glucose fermentation. As transfers are done every 24 hours we expect growth in this last phase to be under weaker selection (but such growth could contribute to biomass production measured at 24 hours).

#### Lag phase

To determine the growth lag, we measured the rate at which glucose was consumed by HPLC. We could not use automated optical density measures (OD, see below), because growth during lag phase occurred below the detection limit of our bioscreen machines. For HPLC, we measured two independently cultured replicates of the ancestral (“Gen0”) haploid, evolved diploid (“Gen1767”), and three haploid and three diploid colonies isolated from 1302 generations (these colonies were a subset of the haploid and diploid colonies isolated from this time point used in all other experiments). For each, a small amount of previously frozen culture was inoculated into 10 mL YPD and grown for 48 hours, shaking at 30

C. Five replicate test tubes were then inoculated with 100
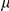
L from each culture. At precisely 2, 4, 6, 8 and 24 hours, one replicate tube for each colony was removed from the incubator. Tubes were thoroughly vortexted and 2 mL aliquots were pelleted. 1 mL of liquid from each tube was filtered with a 25 mm filter into a sterile culture vial. Vials were kept at 4 until the end of the experiment (24 hours). Samples were then run on an Agilent 1100 Series LC/MSD with a Nuleogel Ion 300 OA column at 71

C. The solvent was 4.25 mM H
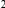
SO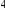, run isocratically at 0.55 ml/min. Glucose was detected and quantified with a refractive index detector running at 40

C, where the reduction of glucose levels during the earliest time points reflects growth during lag phase.

#### Growth Rate

Naively, one might expect that diploid mutants overtake haploids because diploids grow faster during log growth, which we tested in two sets of experiments, one which examined the small number of colonies isolated throughout the time series and a second that examined 20 colonies isolated from 1023 and 1302 generations. Growth rates were determined using the Bioscreen C Microbiological Workstation (Thermo Labsystems), which measures optical density (OD) in 100 well honeycomb plates, with constant shaking and temperature. Previous work had found growth rate can be variable across bioscreen runs (likely due to small differences in medium, A.C. Gerstein, unpublished results). As these two sets of experiments were not conducted at the same time we compare results only within a single bioscreen experiment. Plates were streaked from frozen stock and allowed to grow for 72 hours. An inoculation containing multiple colonies was allowed to grow overnight in 10 mL YPD. 100 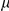
L was transferred into 10 mL of fresh YPD, mixed well, and seven 150 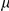
L aliquots from each test tube were placed into different bioscreen wells.

Order of wells was fully randomized. Plates were kept in the Bioscreen C at 30

C, with OD readings taken every 15 minutes for 48 hours. The maximal growth rate was determined for each well as the spline with the highest slope, from a loess fit through log-transformed optical density data (analysis program written by Richard Fitzjohn in the R programming language [15] as previously described [16]). We interpret this slope as the maximum growth rate in each bioscreen well (which we refer to as “growth rate” throughout).

#### Biomass production and number of cells at 24 hours

The ability to convert nutrients in the medium into cellular material may also differ over time or between haploids and diploids. We interpret the optical density (OD) at 24 hours as a measure of total biomass production between transfers. For each bioscreen well, we calculated the optical density at 24 hours minus the optical density at the start of the experiment. As haploid and diploid cells (and cells of different genotypes) may differ in cell size, differences in biomass production do not necessarily correlate to differences in absolute number of cells, and we avoid interpreting them as such. To obtain a measure of the number of cells present at 24 hours we conducted hemocytometer counts of ancestral (Gen0) and evolved (Gen1767) culture, as well as the five haploid and five diploid colonies isolated after 1023 and 1302 generations of evolution. We note that both growth rate and biomass production were measured in a different environment than the original experiment (100 well honeycomb plates versus large test tubes), and it is possible that a different result could have been obtained if we examined these parameters in the evolutionary environment. Experiments in our lab (unpublished results, A.C.G.) have found very little difference between the parameters measured in these different environments, though we did not specifically test the lines of specific interest for this project.

### Competition against a common competitor

A competition experiment was undertaken to gain a comprehensive measure of the ability of each line to compete for resources. A common competitor was created as previously described [17]. Briefly, we inserted a 3320-bp region of the pJHK043 plasmid (generously provided by John Koschwnez, FAS Center for Systems Biology, Harvard University) containing YFP under control of the *ACT1* promoter linked to a histidine marker into the *HIS* locus of BY4741 (*MATa his3* 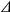 *1 leu2* 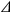 *0 met15* 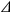 *0 ura3* 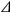 *0*), obtained from Open Biosystems (Thermo Fisher Scientific). Competitive fitness was measured by tracking the ratio over time of fluorescing (competitor) cells to the non-fluorescing cells of interest. We measured the competitive fitness of ancestral and evolved culture, and twenty haploid and diploid colonies isolated from generations 1023 and 1302.

For each line of interest culture was struck from frozen onto YPD plates and grown for 72 hours at 30

C, at which point colonies were inoculated into 10 mL YPD and grown for 48 hours. To start the competition experiment, 100
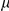
L of the competitor and 100
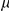
L of the strain of interest were inoculated into 10 mL of YPD. Four replicates were initiated for each line of interest. We performed transfers that exactly mimicked the initial evolution experiment (100 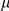
L transferred from each tube after 24 hours of growth into 10 mL fresh medium in large test tubes) for three days. Each day (including the initial day of the experiment), exactly two hours after transfer, 1 mL of culture from each tube was aliquoted into an eppendorf, pelleted, and resuspended in 1 mL of sodium citrate. 150
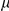
L from each eppendorf was aliquoted into one well of a 96 well plate and immediately run on an LSRII flow cytometer with the High Throughput Sampler attachment. 10000 cells were measured from each well.

Data was analyzed in FlowJo version 8.7 (Tree Star, Inc.). Small debris was excluded with an initial gate then gates were drawn around the two clusters of non-fluorescing and fluorescing cells, by examining plots of FITC-A and AmCyan-A. Clusters were always easily distinguished. The absolute number of cells in each gate for each day of the experiment was determined. The competitive fitness (*m*) was determined for each line using the formula for evolutionary change:
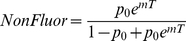
(4)


where *NonFluor* is the fraction of non-fluroescing cells, *p* 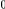 is the initial fraction of non-fluorescing cells at the start of the experiment, T is the generation number (measurements were done on days 0, 1, 2, and 3 which corresponds to 0, 6.7, 13.2, and 20.0 generations) and *m* is the Malthusian parameter of the experimental strain minus that for the YFP-marked competitor (relative growth rate). We use the nls function in the R programming language [15] to determine the best fitting *p* 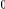 and *m* for each competition assay.

### Direct competition between haploids and diploids

We also directly competed contemporaneous haploid and diploid colonies isolated from the original experiment to test whether diploid colonies isolated at 1302 or 1488 generations (the first generation where only diploid colonies were sampled) were able to outcompete a population of the haploids colonies isolated at 1302 generations. All 24 colonies originally isolated at 1302 and 1488 generations were struck again to single colony from freezer stock maintained at −80

C. A single colony from each was inoculated into 10 mL YPD and allowed to grow overnight. 20
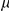
L from each of the six haploid colonies isolated from 1302 generations were combined in a single 10 mL tube and grown together for a second night, forming the haploid competitor. Two diploid populations were similarly created from diploid colonies isolated at 1302 and 1488 generations by inoculating 10
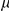
L of 24 hour cell culture from each of the appropriate diploid colonies into 10 mL YPD. The next day, two replicate competitions for each single diploid line and two replicate competitions for each diploid population were initiated by combining 50
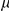
L of the haploid competitor and 50
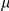
L of diploid culture into 10 mL YPD. 100
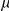
L was transferred into fresh medium every 24 hours for the next 14 days, exactly mimicking the original evolution experiment. The initial (day 0) tubes were kept at 4

C for the duration of the experiment. Day 14 tubes were also kept at 4

C after the completion of the experiment until we were able to assay them as described below.

An assay based on standard yeast lab protocols was developed to differentiate between haploid and diploid cells using hydroxyurea, a drug that arrests yeast cells during DNA synthesis [18]. Our usual flow cytometry protocol measures cells at all stages of the life cycle, thus haploid cells in G2 have the same DNA content as diploid cells in G1. Arresting cells in S phase, however, allows us to discriminate between haploid and diploid colonies. Seven days after the last transfer, 100
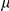
L from all day 0 and day 14 tubes were transferred into fresh medium and grown overnight. The next day, 100
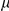
L from each tube was again transferred into fresh medium and allowed to grow for four hours. 1 mL from each tube was then added to 200
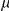
L of 1 M hydroxyurea and transfered into eppendorfs. Eppendorfs were laid flat in a shaking 30

C incubator for 3 hours. We then used the flow cytometry protocol previously described to assess ploidy using a FACSCalibur [2]. Culture was pelleted, resuspended in cold 70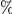 ethanol, and kept at room temperature overnight. The next day culture was pelleted and resuspended twice in 1 mL sodium citrate, 25
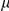
L RNAse A was added and tubes were incubated at 37

C overnight. Tubes were again pelleted and resuspended twice in 1 mL sodium citrate. 30
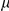
L sytox green was added and tubes were left at room temperature in the dark overnight. The next morning all samples were run on a FACSCalibur. Although this method does not perfectly assay ploidy level (some cells escape arrest, see Figure S3) we found the fraction of un-arrested cells to be fairly consistent. We focus on the the change in the frequency of diploid cells initially and after 14 days of competition, so we do not think our results should be biased towards finding an increase in either ploidy level.

### Replicate evolution experiment

Lastly, we re-evolved cultures maintained in the freezer to determine if we could recapitulate the original result of diploid takeover. We re-evolved culture revived from 1302 generations. We also initiated a second set of tubes where we spiked in a small number of 1488 generation diploids (i.e., the first time point after diploidy had swept) alongside the 1302 generation culture. We thawed completely the freezer tubes of the entire population that had been frozen after 1302 and 1488 generations during the initial evolution. 20 ul aliquots from generation 1302 were inoculated into 10 mL test tubes of YPD for the single time point replicates, while 18 uL of culture isolated at 1302 generations was combined with 2 uL of culture from 1488 generations for the mixed evolution replicates. Cultures were grown exactly as in the original experiment with 1∶101 dilutions in 10 mL YPD every day. In the first block of the experiment we re-restarted 20 test tubes from Gen1302 culture. The initial (day0) tubes were maintained at 4

C for the duration of the experiment; after 26 days of transfers we transferred 100 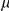
L of both day0 and day26 tubes into 10 mL fresh YPD and allowed them to grow overnight. We then sampled the proportion of haploids and diploid from tubes using the same FACSCalibur protocol described above. A second block of the experiment was then initiated. We continued the initial tubes for 15 additional days. We also started a second set of evolution tubes; 10 new tubes were started from 1302 generation culture as well as 20 replicate tubes spiked with cells from 1488 generations. Statistical analyses was conducted to account for a block (or length of experiment) effect. We first compared the two blocks of evolution started from culture isolated at 1302 generations (41 days evolution vs. 15 days of evolution). We then compared the replicates started with 1302 generation culture against those started with 1302+1488 generation culture. A Fishers' exact test was used to test whether there was a significant increase in diploidy for each experiment. We tested for a difference between experiments (pure Gen1302 culture vs. Gen1302+Gen1488) using a two-way t-test.

## Results and Discussion

We sought to determine why diploids were able to overtake haploids during an 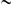
1800 generation batch culture evolution experiment [2]. We have focused on one of five lines independently evolved in YPD (“Line A”) that showed this pattern. By isolating many haploid and diploid colonies from throughout the initial evolution experiment we were able to assay fitness of colonies of different ploidy that were present in the same population. We previously found the ancestral strain to be aneuploid for chromosome IX [19]; though we have not tracked this aneuploidy directly in the experiments presented here, we found no evidence of aneuploidy for any chromosome in 10 colonies (5 haploid and 5 diploid) isolated at 1302 generations (Figure S4). Variation for genome size was found by flow cytometry at several intermediate timepoints (Figure 1). We used the kmeans function in the R Programming Language [15] to assign each colony to a cluster, with the number of clusters (k) set to 2 (k = 2 significantly decreased the within group sum of squares). As cluster assignments correspond to haploid and diploid genome sizes based on control samples, we refer to all colonies in the first cluster as haploids and colonies in the second cluster as diploids. The first diploid colony was sampled at 744 generations and the last haploid was sampled at 1302 generations, thus polymorphism for genome size was maintained for at least 558 generations.

**Figure 2:**
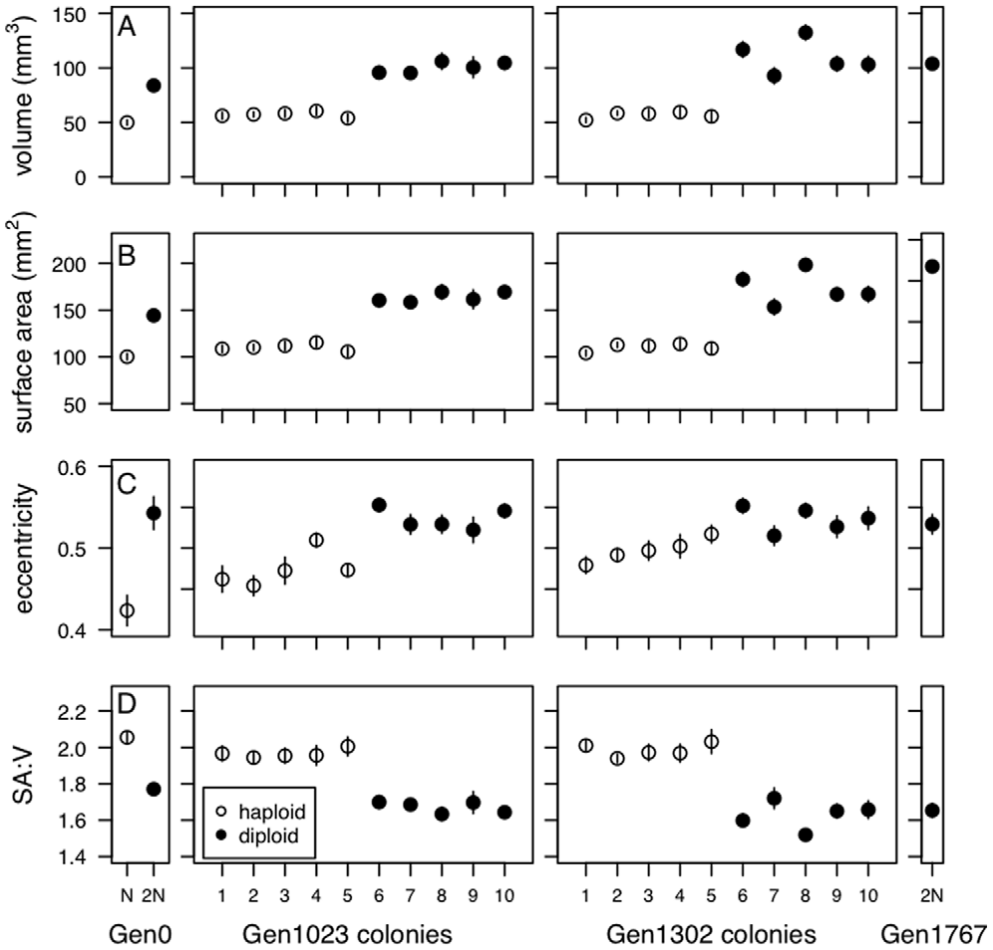
Cell size and shape at 1023 and 1302 generations. Cell size (A: volume, B: surface area) and shape (C: eccentricity, D: surface area to volume ratio) of ancestral and evolved populations, as well as five haploid and five diploid colonies isolated at 1023 and 1302 generations. Numbers assigned to a colony are used consistently throughout all assays (and in all figures).

**Figure 3:**
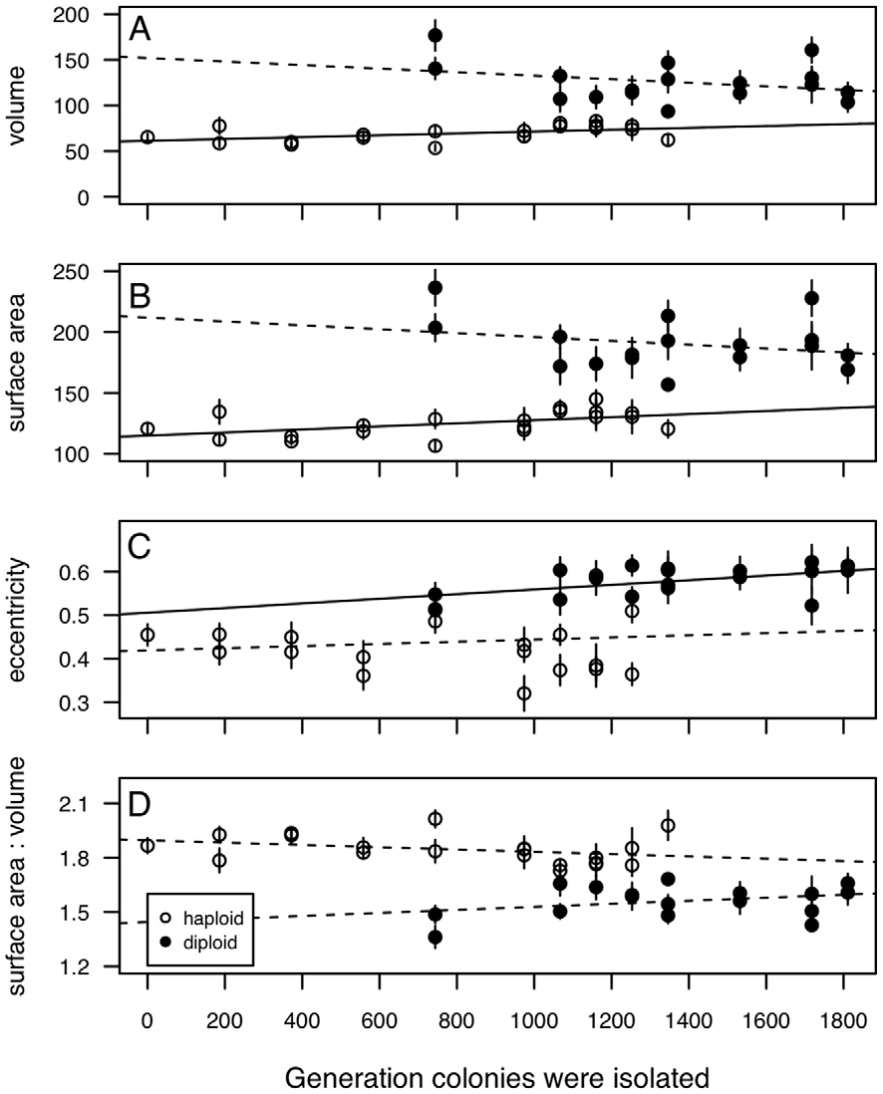
Cell size and shape across the time series. Cell size (A: volume, B: surface area) and shape (C: eccentricity, D: surface area to volume ratio) measures for 15 cells from two to four colonies isolated at each time point. Haploid and diploid colonies were analyzed separately to test the relationship between cell size/shape and generation of colony isolation. Solid lines indicate a a significant linear regression (*p* 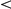
0.05) while dashed lines indicate a nonsignificant trend (*p* 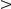 0.5). Here and in later figures, error bars represent 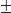 1 SE.

We found consistent differences in cell size and shape between haploid and diploid colonies. Haploid cells isolated at both 1023 and 1302 generations had significantly smaller volumes and surface areas than diploid cells (Table 1, Figure 2A, B), as is commonly observed [7], [20]. As predicted based on the equations for cell shape, diploid cells were also more eccentric (i.e., less spherical) and had a significantly lower surface area to volume ratio than haploid cells (Table 1, Figure 2C,D; see also [15]). Interestingly, we found evidence that cell size and shape may have changed within a ploidy level across the time frame of our initial experiment. As shown in Figure 3, we found a significant increase in both cell volume and surface area of haploid cells over time (using the lm function in the R programming language [15]); volume: F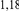 = 5.87, *p* = 0.026, surface area: F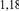 = 6.48, *p* = 0.02), with no significant change in eccentricity (F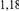 = 0.35, *p* = 0.56) or surface area:volume ratio (F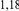 = 2.27, *p* = 0.15). The only significant change for diploid colonies isolated at many time points was eccentricity; diploid cells became more elongated over time (eccentricity: F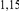 = 5.58, *p* = 0.032; volume: F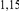 = 1.59, *p* = 0.23; surface area: F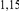 = 1.09, *p* = 0.31; surface area:volume: F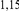 = 1.78, *p* = 0.21). An adaptive increase in cell size has previously been found for *E. coli* when evolved in minimal medium for 2000 generations under similar batch culture conditions [21]. As previously mentioned, cell volume alone may contribute to potential differences between cells of differing ploidy [9]. Cell volume is highly correlated with surface area, eccentricity and surface area:volume whether we compare across the time series or within Gen1023 or Gen1302 colonies (statistics are presented in Supplementary Tables S1 and S2). We thus focused only on cell volume as a potential correlate with growth phase components and fitness correlates of haploid and diploid colonies.

**Table 1:** Cell size and shape statistics.

	full time series	1023 generations	1302 generations
**volume**	t 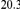 = −10.21 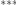	t 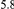 = −17.7 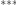	t 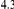 = −7.6 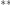
(N)	69.5 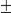 1.4	57.3 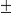 1.2	56.8 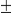 1.5
(2N)	126.2 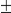 3.2	100.5 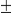 2.5	109.8 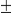 7.6
**surface area**	t 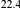 = −11.8 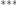	t 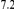 = −18.9 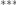	t 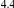 = −8.0 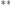
(N)	124.9 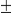 3.2	110.3 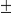 1.8	110.3 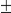 2.0
(2N)	190.8 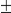 1.6	163.9 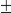 2.6	173.8 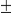 8.7
**eccentricity**	t 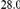 = −7.5 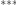	t 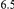 = −5.5 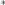	t 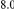 = −4.1 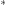
(N)	0.578 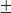 0.008	0.449 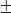 0.021	0.495 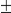 0.014
(2N)	0.436 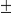 0.008	0.572 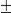 0.013	0.570 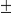 0.015
**surface area:volume**	t 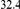 = 10.4 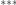	t 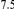 = −16.8 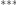	t 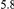 = −9.5 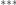
(N)	1.84 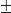 0.012	1.97 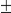 0.012	1.99 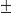 0.018
(2N)	1.56 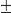 0.014	1.67 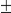 0.016	1.63 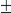 0.038

Cell size & shape statistics comparing 20 haploid and 17 diploid colonies isolated across the full time series (haploid colonies isolated between 0 and 1346 generations, diploids between 744 and 1811 generations), and five haploid and five diploid colonies isolated at each of 1023 and 1302 generations of evolution. In each case we compared haploid and diploid colonies using a Welch two sample t-test, not assuming equal variance;

***: *p* 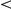
0.0001,

**: *p* 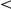 0.001,

*: *p* 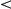 0.01.

**Figure 4:**
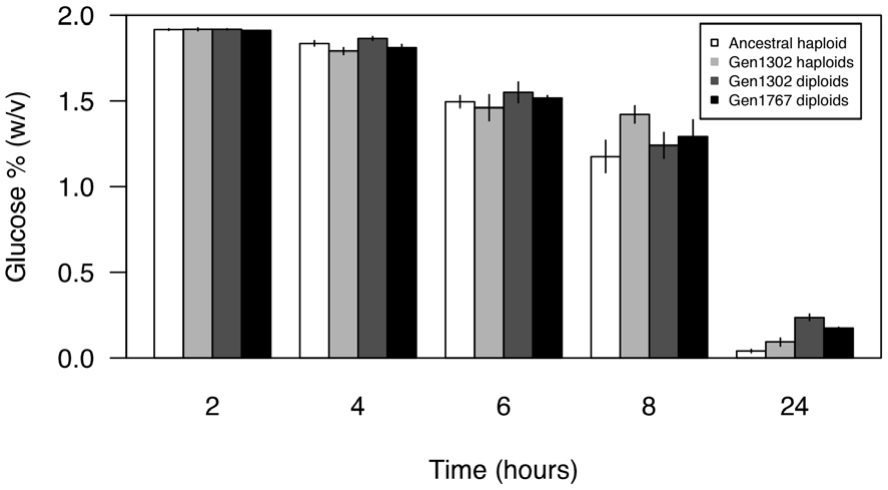
Lag phase fitness proxy. Glucose % (w/v) measured by HPLC post transfer into new medium. Haploids (isolated at the first time point and after 1302 generations of evolution) and diploids (1302 generation and 1767 generation) do not differ significantly in the amount of glucose present in the medium at the early time points, suggesting that their growth lags do not differ substatially.

Three different growth phase components were measured in an attempt to capture the primary phases of growth experienced by yeast cells in the 24 hours between transfers during the original experiment [?]. Surprisingly, we found that none of these component fitness measures indicated an advantage to diploidy, despite diploids overtaking haploids. To test whether the lag phase of growth differed between haploids and diploids, we used mass spectrometry to measure the percentage of glucose remaining in the medium every 2 hours until 8 hours after transfer for four populations initiated from colonies isolated at 0 (haploids), 1302 (haploids and diploids), and 1767 generations (diploids). We also measured the percentage of glucose remaining in the medium at 24 hours. If lag phase differs between haploids and diploids, we expect to find differences in the glucose remaining at the early time points. As the amount of glucose present initially is the same, any difference in lag phase would be recovered as a difference in the amount of glucose remaining in the medium due to differences in the rate of glucose metabolism. However, as shown in Figure 4, a two-way ANOVA indicated that although the percentage of glucose decreases in the medium as post-transfer time increases (F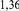 = 1359.8, *p* 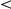
0.0001), no differences were detected between haploid and diploid colonies (F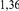 = 0.82, *p* = 0.37), nor was there a significant interaction between generation and ploidy (F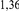 = 1.80, *p* = 0.19). Similarly, when we compared the four populations, ploidy did not significantly affect glucose % over the first eight hours of growth (t-test; 2 h: t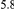 = 5.7, *p* = 0.7; 4 h: t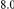 = −1.5, *p* = 0.2; 6 h: t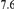 = −1.1, *p* = 0.3; 8 h: t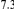 = 0.69, *p* = 0.5). These results indicate that haploid and diploid colonies begin to grow and utilize glucose at similar rates, suggesting no difference in lag phase. Interestingly, after 24 hours diploid lines had significantly more glucose remaining in the medium than haploid lines (t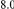 = −5.4, *p* = 0.0007), suggesting that they are either less efficient at utilizing glucose for growth and biomass production or that they switch to metabolizing ethanol before glucose is used up.

**Figure 5:**
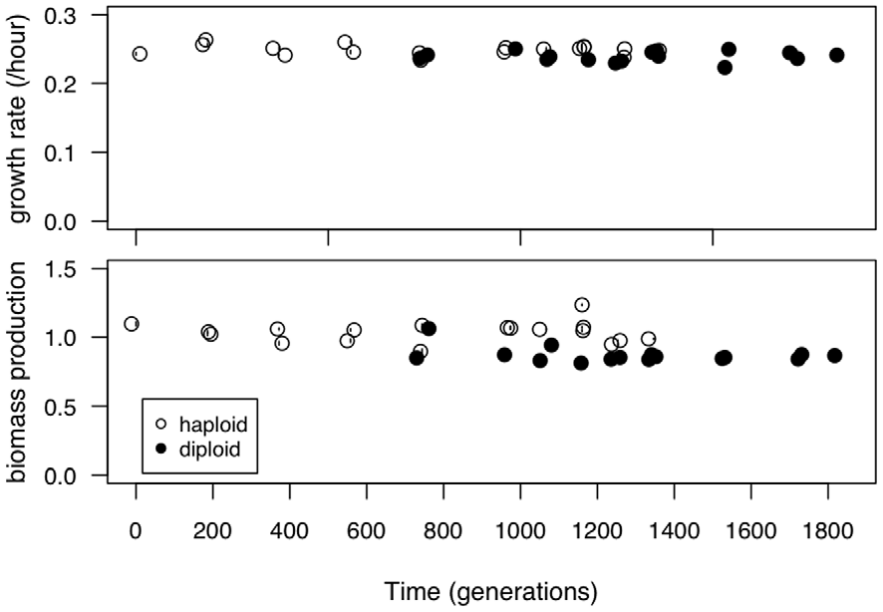
Growth rate and biomass production across the time series. Growth rate and biomass production were measured for 20 haploid and 17 diploid colonies isolated throughout the original experiment. Points are plotted with slight jitter on the x-axis for viewing purposes.

Looking across the entire time series (Figure 5), neither growth rate nor biomass production predicted why diploid colonies might be able to invade haploids. Using a partial correlation test to remove the effect of time, haploid colonies both grew faster (t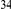 = −2.87, *p* = 0.004) and reached higher biomass (t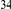 = −5.46, *p* 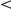 0.0001) than diploid colonies. The correlation between time and growth rate was not significant for either ploidy level (haploids: r = −0.14, t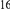 = −0.59, *p* = 0.57; diploids: r = 0.01, t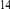 = 0.05, *p* = 0.96) nor time and biomass production (haploids: r = 0.02, t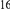 = 0.07; *p* = 0.94, diploids: r = −0.41, t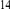 = −1.68, *p* = 0.11).

**Figure 6:**
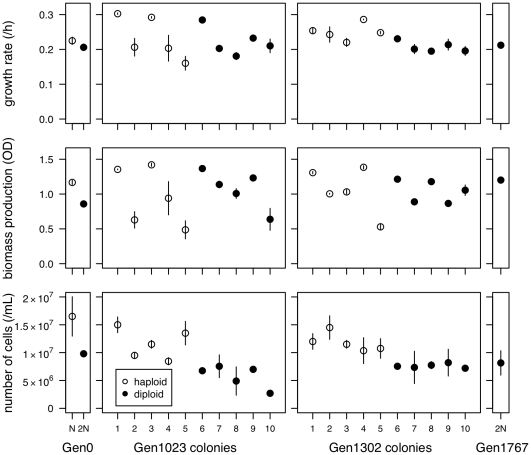
Growth rate, biomass production and the number of cells at 24 hours. Growth rates (top), biomass production (middle) and the density of cells (bottom) measured for ancestral haploid and diploid lines, five colonies of each ploidy after 1023 generations and 1302 generations of evolution, and the diploid population after 1767 generations.

We then looked in greater depth at the populations from 1023 and 1302 generations. Diploid colonies had a lower growth rate than haploid colonies, significantly so at 1302 generations (Figure 6; Welch's two-sample t-test; 1023: t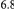 = 0.33, *p* = 0.75; 1302: t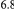 = 3.41, *p* = 0.01). Biomass production also did not differ significantly between haploid and diploid colonies when they were isolated at the same time point (1023 generations: t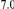 = −0.491, *p* = 0.64; 1302 generations: t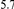 = 0.07, *p* = 0.95). Neither growth rate nor biomass production showed a significant correlation with cell volume for colonies isolated across the time series when we control for ploidy using a partial correlation (growth rate: *p* = 0.84; biomass production: *p* = 0.08) or when we examine colonies from 1023 and 1302 generations together (growth rate: *p* = 0.80; biomass production: *p* = 0.52). Although researchers typically assume that growth rate is the primary factor under selection in batch culture [22], our results do not support this. One caveat to this conclusion is that, because of the large number of colonies assayed in replicate, these parameters were measured in 100 well honeycomb plates rather than in the test tubes of the original experiment; it is possible that growth rate differences might have been apparent in a different environment.

When we measure population size after 24 hours (Figure 6), we found that diploid genotypes produce significantly fewer individuals within a growth cycle than haploids. Population size correlated very strongly with cell volume (t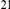 = −5.68, *p* 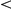
0.0001), though this relationship is driven entirely by ploidy, as there is not a significant correlation when we use a partial correlation to control for ploidy (*p* = 0.301) The fact that glucose consumption appears to be equal between haploid and diploid populations despite fewer individual diploid cells indicates that the average diploid individual metabolizes glucose faster than the average haploid individual. The differences we found in cell size and shape likely explain how diploid cells (which are fewer in number, but larger) are able to consume glucose at the same overall rate as haploid cells.

Selection may actually favour a slower growth rate if there is a tradeoff between growth rate and a second fitness component. For example, Blount *et al*. [23] found that mutant *E. coli* that have acquired the ability to metabolize citrate outcompete individuals that cannot; citrate mutants have a significantly slower growth rate and a longer lag phase than other individuals isolated at the same time, yet they reach much higher optical density. Novak *et al.* [24] explicitly tested for a tradeoff between growth rate and yield (biomass production) in 12 *E. coli* populations that had evolved for 20 000 generations. They did not find a significant tradeoff when comparing across the 12 populations with samples isolated at multiple time points. Interestingly, however, they do find evidence for significant tradeoffs when they look at many colonies isolated from the same population at one time point. We, however, do not find evidence for a negative tradeoff in our experiment, at least between growth rate and biomass production. When we compare across all timepoints (Figure 5), we find a significant positive correlation between growth rate and yield (t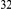 = 3.75, *p* = 0.0007, r = 0.55), yet this relationship is largely driven by the effect of ploidy. If we test for a correlation within haploid or diploid populations we find no significant correlation for either, but in both cases the correlation is positive (haploid: t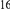 = 0.83, *p* = 0.42, r = 0.20; diploid: t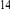 = 0.95, *p* = 0.36, r = 0.25). Our findings are similar to results obtained by [25] when examining *S. cerevisiae* clones isolated during 260 generations of growth in a chemostat. Specifically, Adams *et al.* found that growth rates changed very little over the course of the experiment and that the growth rate of some of their later time point clones is lower than the initial clones. It thus seems that neither growth rate nor biomass production are able to explain why diploids overtook haploids in our evolution experiment.

Fitness assays that examine population level parameters may not capture the dynamics that occur when different genotypes (or cells of differing ploidy) are in direct competition with each other, either because of interactions between individuals of different ploidy levels or because of unmeasured components of fitness in batch culture. For example, previous work on *Candida albicans* did not find a significant correlation between fitness measured by direct competition experiments and fitness measured on isolated populations (examining either growth rate or stationary phase density; [26]). We thus turned our attention to competition assays that account for interactions between different ploidy types and that integrate fitness across the entire 24 hour batch culture cycle.

**Figure 7:**
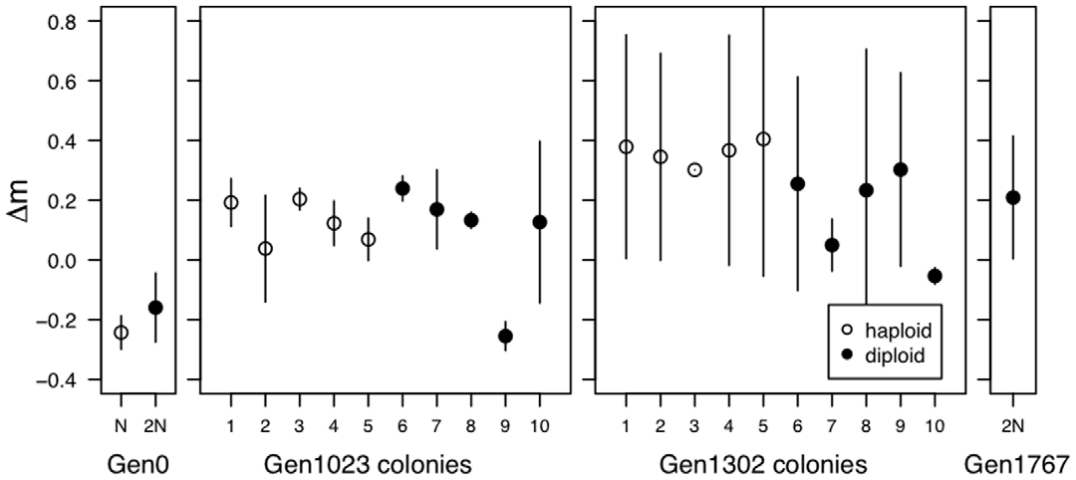
Competition against a common competitor. Haploid and diploid colonies were competed directly again a common marked competitor for 72 hours. The y-axis (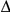
m) is the difference in malthusian growth rate between the given strain and the common competitor.

We first determined the competitive ability of generation 0 haploid colonies and generation 1767 diploid colonies, as well as haploid and diploid colonies isolated at 1023 and 1302 generations, against a closely-related marked competitor (both our ancestor and the common competitor are derivatives of *S. cerevisiae* strain S288C). This assay was, however, also unable to explain why diploids were able to overtake haploid colonies (Figure 7). Altogether, we found that only the generation at which a colony was isolated significantly affected competitive ability (two-way ANOVA, time: F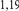 = 22.9, *p* = 0.00013, ploidy: F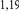 = 3.18, *p* = 0.091, time*ploidy: F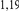 = 3.21, *p* = 0.089). When we look at the difference in competitive ability between colonies of different ploidy isolated at 1302 generations, we find that haploids compete significantly better than diploid colonies (two-way t-test: t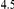 = 2.88, *p* = 0.039), although ploidy did not significantly affect competitive ability among the 1023 generation colonies (two-way t-test: t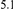 = 0.458, *p* = 0.666).

**Figure 8:**
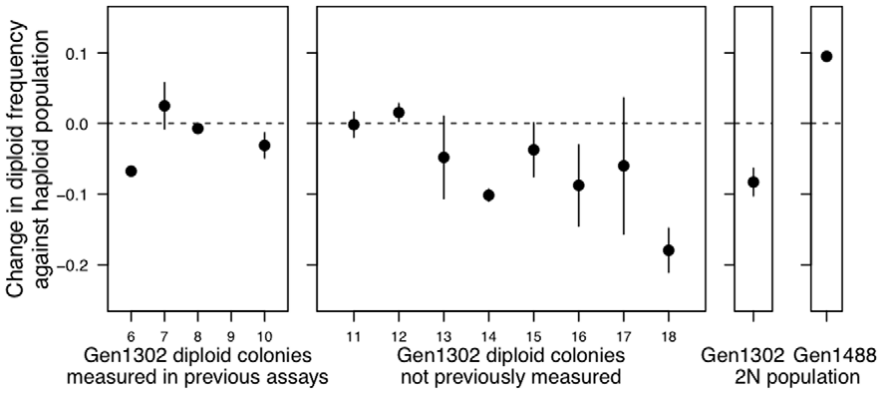
Competition against the haploid population from 1302 generations. Thirteen diploid colonies isolated at 1302 generations, a pool of 12 1302 generation diploids (“Gen1302 2N population”) and a pool of 24 diploid colonies isolated at 1488 generations (“Gen1488 2N population”) were competed against a population of the 6 haploid colonies isolated at 1302 generations. Contamination arose in one of the diploid colony competitions, and we were unable to measure the results of this competition (the blank space in the first panel). Only the population of diploids from 1488 generations (the first time point after diploidy swept in the original experiment, rightmost panel) was consistently able to outcompete the haploid population. All competitions were started at 50:50 (v/v) with transfers into fresh medium every 24 hours for 14 days. Standard error bars based on two replicate competitions.

Experiments that compete colonies against a common competitor (or the ancestor) also do not precisely mimic the original evolution experiment, however. If non-transitive fitness changes are occurring, comparing fitness against the ancestral type does not inform us about competitive ability against the actual genotypes that were present at any point in time. Such non-transitive fitness interactions have been shown to be important in some previous microbial experiments [27], but not others [28]. To control for this potentially important factor, the best fitness assay is one that directly competes colonies from the same time point together. We thus competed a population of 6 haploid colonies isolated at 1302 generations against single diploid colonies isolated at 1302 generation (for 12 individual diploid colonies), as well as against a pooled populations of 12 diploids colonies isolated from 1302 generations (“Gen1302 2N population”) and a pool of 24 diploid colonies isolated at 1488 generations, after diploids appeared to have fixed (“Gen1488 2N population”). As we are primarily interested in the ability of diploids to overtake haploids (as was observed in the original experiment), we conducted a one-way t-test to look for a significant increase in diploid frequency after 14 days of competition. As shown in Figure 8 & Table 2, only the population of diploids isolated at 1488 generations, i.e., the first generation where only diploid colonies were sampled, were significantly able to increase in diploid frequency. None of the single diploid colonies nor the population of diploids created by combining single colonies from 1302 generations significantly increased in frequency compared to the haploid population from 1302 generations (Table 2; one of the previously assayed diploid lines became visibly contaminated during the experiment and was not assayed, “colony 4” in the first panel of Figure 8).

**Table 2:** Competition against haploid population from 1302 generations.

competitor	one-way t-test
1302gen - colony 6	t 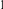 = −12.3, *p* = 0.97
1302gen - colony 7	t 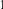 = 0.8, *p* = 0.29
1302gen - colony 8	t 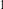 = −1.14, *p* = 0.77
1302gen - colony 9	contaminated
1302gen - colony 10	t 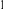 = −1.7, *p* = 0.83
1302gen - colony 11	t 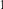 = −0.1, *p* = 0.53
1302gen - colony 12	t 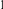 = 1.21, *p* = 0.22
1302gen - colony 13	t 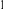 = −0.8, *p* = 0.72
1302gen - colony 14	t 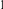 = −11.8, *p* = 0.97
1302gen - colony 15	t 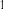 = −1.0, *p* = 0.75
1302gen - colony 16	t 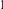 = −1.5, *p* = 0.82
1302gen - colony 17	t 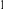 = −0.6, *p* = 0.68
1302gen - colony 18	t 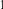 = −5.7, *p* = 0.94
1302gen - 2N population	t 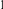 = −4.2, *p* = 0.93
1488gen - 2N population	t 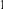 = 15.0, *p* = 0.02

t-test results of single diploid colonies isolated from 1302 generations and diploid populations from 1302 and 1488 generations competed directly against a population of haploids isolated from 1302 generations. Colony ordering as in Figure 8; the first 5 colonies are the same five colonies measured in the other fitness experiments. The assay compares the frequency of diploid cells after 14 days of competition using a FACSCalibur.

In summary, none of our fitness assays predicts that diploid colonies isolated from either 1023 or 1302 generations would be able to overtake contemporaneous haploids present in the environment at the same time point during the original experiment. Rather, our results indicated that the fitness advantage that allowed eventual takeover by diploids arose in only a subset of diploid lineages, which predominated by 1488 generations. Recall, however, that growth rates were not significantly higher at the end of the experiment (Figure 6, Gen1767), nor were they significantly higher at generation 1488 (Figure S5), leading us to conclude that growth rate measures fail to predict competitive advantage in these diploids.

**Figure 9:**
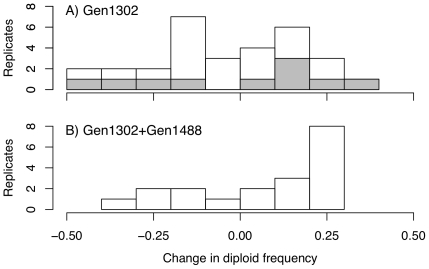
Replicate evolution experiment. The experimental evolution study was restarted from A: culture from 1302 generations and B: culture from 1302 generations spiked with 5% diploid culture from 1488 generations. Replicates were evolved through batch culture in exactly the same way as the original experiment [?]. The 1302 generation culture was evolved for 41 days (20 replicates) and 14 days (10 replicates - shaded bars in top panel), mixed culture was evolved for 14 days (20 replicates).

To further explore the population dynamics of the population isolated at 1302 generations, we re-evolved freezer culture that was acquired during the original experiment. Culture isolated after 1302 generations of evolution was re-evolved during two blocks, one that lasted for 41 days and one that lasted 15. There was no significant difference in the total change in diploid frequency between blocks (t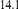 = −0.35, *p* = 0.73), thus we combine them for analysis. Across 30 replicate tubes initiated from a population sample taken from 1302 generation culture, there was no significant change in diploid frequency (t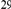 = −0.94, *p* = 0.35), with 14 tubes showing an increase in diploid frequency and 16 tubes showing a decrease (Figure 9A), with tremendous variation among replicates. The set of replicates initiated with 18 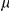
L culture isolated after 1302 generations spiked with 2 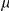
L culture from 1488 generations more often exhibited an increase in diploid frequency, but the change in diploid frequency was again not significantly different than 0 (Figure 9B: 13 test tubes increased in diploid frequency, 6 decreased; t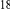 = 1.4, *p* = 0.17). The starting diploid frequency was not significantly different between the two treatments (t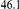 = −0.127, *p* = 0.90), yet diploid frequency did increase significantly more when 1302 generation culture was spiked with 5% 1488 generation culture then when it was not (one-way Welch t-test: t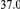 = −1.70, *p* = 0.049).

### Conclusions

The experiments described above aimed to determine how diploid individuals were able to rise in frequency within the ancestral population of haploids. Our results failed to find any fitness advantage of early-arising diploids (generations 1023 and 1302) over haploids. How could the diploids have risen to intermediate frequency without a fitness advantage? Several possible explanations remain. The environments might have been slightly different than in the initial experiments. Alternatively, even though we sampled five diploid colonies at both of these two time points, perhaps we were unlucky and sampled particular unfit diploids. More likely, we note that none of these standard fitness assays would have revealed a fitness advantage if such an advantage is negative frequency dependent. Competition assays starting at different initial frequencies of diploids suggests that the diploids from generations 1023 and 1302 are able to spread when rare, but this competitive advantage declines with frequency (A.C.G., in prep). We hypothesize that this is why the standard fitness measures used here failed to explain the initial rise in diploid frequency.

Our results indicate that there is not a fitness-related trait that uniformly differs between haploids and diploids and that allows diploids to overtake haploids whenever they appear. Rather, we conclude that only a subset of diploids, which predominated late in the experiment (generation 1488), are competitively superior and capable of fixing within the population (Figure 6). Our direct competition assay and replicate evolution experiment both suggest that the diploids we sampled (specifically, diploids colonies isolated at 1302 generations) are unlikely to be the same exact diploid genotypes that overtook the haploid population during the initial experiment. Interestingly, fitness measures from later generations failed to show evidence of higher growth rates or biomass production (Figures 4, Figure S1), although diploids from these later generations exhibit competitive superiority (Figures 8 and 9). Current sequencing efforts aim to identify the causative mutations underlying the advantage of later generation diploids. We could then determine whether the mutation was accessible or beneficial to diploids alone, explaining the consistent conversion of haploid to diploid populations.

The exact selective forces acting within our experiment remain largely unknown. It may be that organisms are adapting to an aspect of the medium (YPD), to the test tube environment (e.g., low oxygen), or to batch culture (i.e., repeated feast and famine). One clue, however, might be that the smaller haploid cells significantly increased in size (approximately 1% increase in volume over 1302 generations). An adaptive increase in cell size has also been found for *E. coli* when evolved in minimal medium under similar batch culture conditions [21]. We hope that future experiments and sequencing efforts will help shed light on this question.

We are left to conclude that the evolutionary dynamics of this system are more complicated than expected, and that none of the standard assays used to measure fitness demonstrate diploid superiority over haploidy across all diploid lines. The picture that emerges is that the ploidy level of any given colony isolated from a particular time point is not the determining factor in whether that individual has high fitness and will spread. We find tremendous trait variation among colonies of the same ploidy level for the majority of traits measured, and the variation among colonies of the same ploidy is often larger than the variation between ploidy levels (e.g., Figure 5). If anything, haploid cells appear to have the higher fitness (for growth rate and biomass) at intermediate time points when both haploids and diploids are present. As Adams *et al*. [25] noted at the end of their paper examining a chemostat-evolved population of *S. cerevisiae* twenty five years ago:

*“The emerging picture of adaptation in such populations, therefore, is that a number of different cell phenotypes may exhibit increased fitness and that the selection of any one of them is unpredictable, depending on the random nature of the mutational events involved. [We believe] a single optimal phenotype may not exist even for simple constant laboratory environments.”*

Although the role of ploidy in our previous evolution experiment [2] seems to be deterministic in that diploids eventually outcompeted haploids in all ten of our replicate lines, ploidy is not the most important differentiating character among cells present in the population. These experiments demonstrate the utility of maintaining a fossil record during batch culture evolution, allowing us to reconstruct the history of selection.

## Supporting Information

Figure S1 **Ploidy polymorphism was measured approximately every 93 generations (14 days) using flow cytomety.** Freezer culture frozen down from the initial evolution experiment was inoculated straight into 10 mL of YPD and grown for 48 hours. We then used hydroxyurea to synchronize the cell cycle and measured 30 000 cells each time point. This assay provides us with a snapshot of ploidy transition from a haploid population at generation 0 to a diploid population after generation 1395. Throughout, there is a second smaller peak at double the current ploidy level due to some cells remaining in the G2 phase (see Figure S3).
(TIFF)

Figure S2 **Representative images of haploid (A) and diploid (B) cells used in imaging experiment.** Elipses were manually drawn around cells to measure the major and minor axes for use in volume, surface area and eccentricity calculations.
(TIFF)

Figure S3 **HU arrested haploid and diploid populations.** Hydroxyurea is used to synchronize the cell cycle of populations. Presented are the measurement of 30 000 cells from a population composed entirely of haploids (black) and 30 000 cells from a population of diploids (grey). This method is not perfect, as some cells escape arrest. We have found the fraction of un-arrested cells to be fairly consistent, however, and as we focus our results on the difference between the ratio of haploids/diploids from one time point to another, this should not bias our conclusions.
(TIFF)

Figure S4
**Relative chromosome coverage.** From generation 1302, genomic DNA from five haploid and five diploid strains was extracted [1] and sequenced in 100 bp single-end fragments using Illumina's HighSeq 2000. Library preps followed standard Illumina protocols (2011 Illumina, Inc., all rights reserved), with each strain individually barcoded. The resulting genomic sequence data were processed using Illumina's CASAVA-1.8.0. Specifically, configureBclToFastq.pl was used to convert to fastq and separate the sequences by barcode (allowing one mismatched basepair). configureAlignment.pl was then used to align each sequence to the yeast reference genome (scergenome.fasta downloaded from the Saccharomyces Genome Database, http: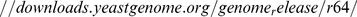). Finally configureBuild.pl was used to obtain coverage data. Average coverage per mapped site was 69.9 across the strains (with a minimum coverage per site of 16.3 for strain colony 4 from 1302 generations). Plotted for each strain is the proportion of sequenced sites from each chromosome relative to the proportion of known mapped sites on that chromosome within the reference genome. Although differences in ploidy cannot be detected with this method, whole-chromosome aneuploids would lead to larger shifts than observed (e.g., an additional chromosome should lead to 2x coverage in haploids and 1.5x coverage in diploids). We conclude that these strains are not aneuploids for whole chromosomes, including the chromosome IX aneuploidy that characterized their founding strain [2]. (The excess coverage on chromosome XII was also observed in an independent sequencing analysis of two strains from the knock-out deletion set, suggesting a common indel of regions on this chromosome or a mapping artefact.)(TIFF)

Figure S5 **Fitness components of 1488 generation diploids do not predict diploid advantage.** Growth rate and biomass production from 10 colonies (5 haploid and 5 diploid) isolated at 1302 generations and 24 diploid colonies isolated from 1488 generations were measured on a Bioscreen C Microbiology Workstation (Thermo Labsystems). Although only diploid colonies were present at 1550 generations, these fitness components do not predict a diploid advantage over the haploid colonies (first panel) that were present immediately before diploid takeover.
(TIFF)

Table S1 **Cell volume correlates strongly with surface area, eccentricity and surface area:volume across colonies isolated across the time series.**
(PDF)

Table S2 **Cell volume correlates strongly with surface area, eccentricity and surface area:volume across colonies isolated at generations 1023 & 1302.**
(PDF)
