# Supplementary figures and images for: Cryptic Fitness Advantage: Diploids Invade Haploid Populations Despite Lacking Any Apparent Advantage as Measured by Standard Fitness Assays
Source: PLoS One. 2011 Dec 9;6(12):e26599. doi: 10.1371/journal.pone.0026599 (PMC3235103; doi:10.1371/journal.pone.0026599)

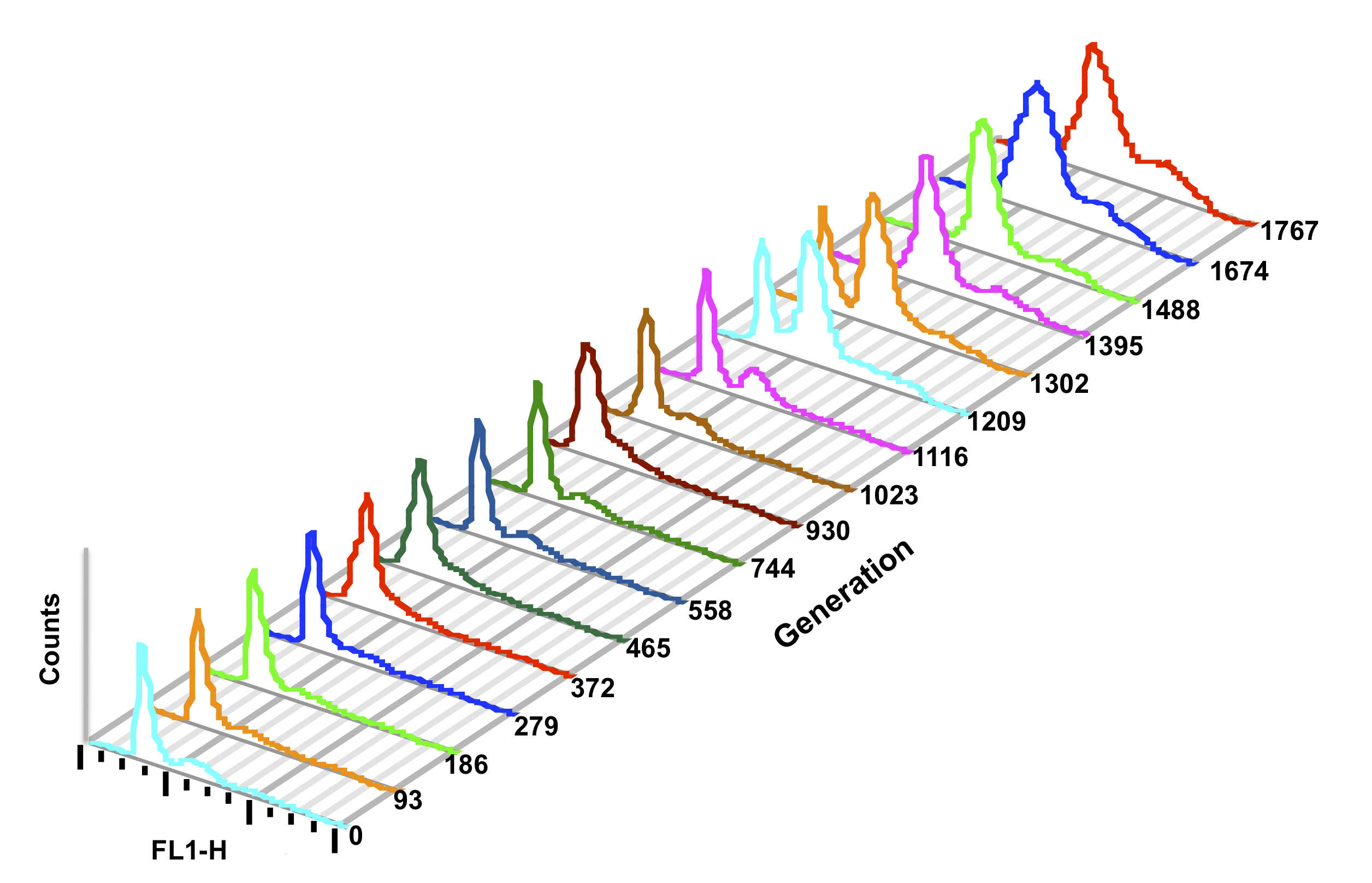

Supplement: Figure S1 — Ploidy polymorphism was measured approximately every 93 generations (14 days) using flow cytomety. Freezer culture frozen down from the initial evolution experiment was inoculated straight into 10 mL of YPD and grown for 48 hours. We then used hydroxyurea to synchronize the cell cycle and measured 30 000 cells each time point. This assay provides us with a snapshot of ploidy transition from a haploid population at generation 0 to a diploid population after generation 1395. Throughout, there is a second smaller peak at double the current ploidy level due to some cells remaining in the G2 phase (see Figure S3). (TIFF) [file pone.0026599.s001.tif]

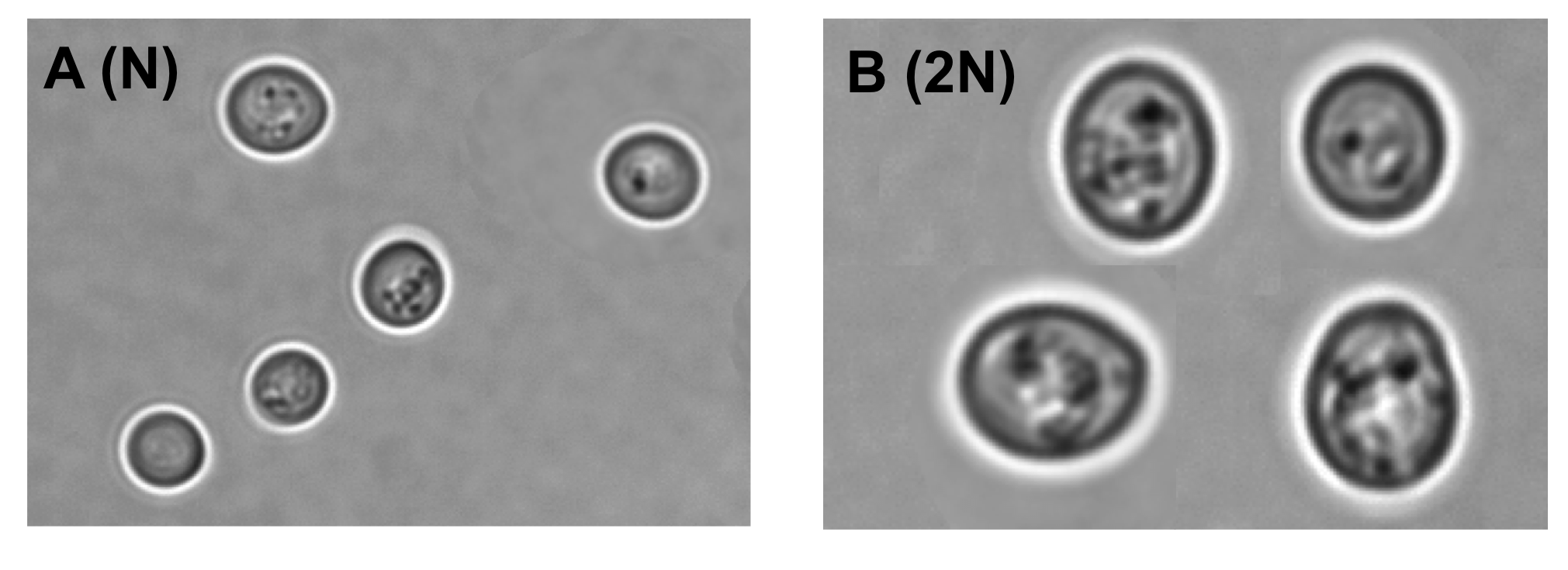

Supplement: Figure S2 — Representative images of haploid (A) and diploid (B) cells used in imaging experiment. Elipses were manually drawn around cells to measure the major and minor axes for use in volume, surface area and eccentricity calculations. (TIFF) [file pone.0026599.s002.tif]

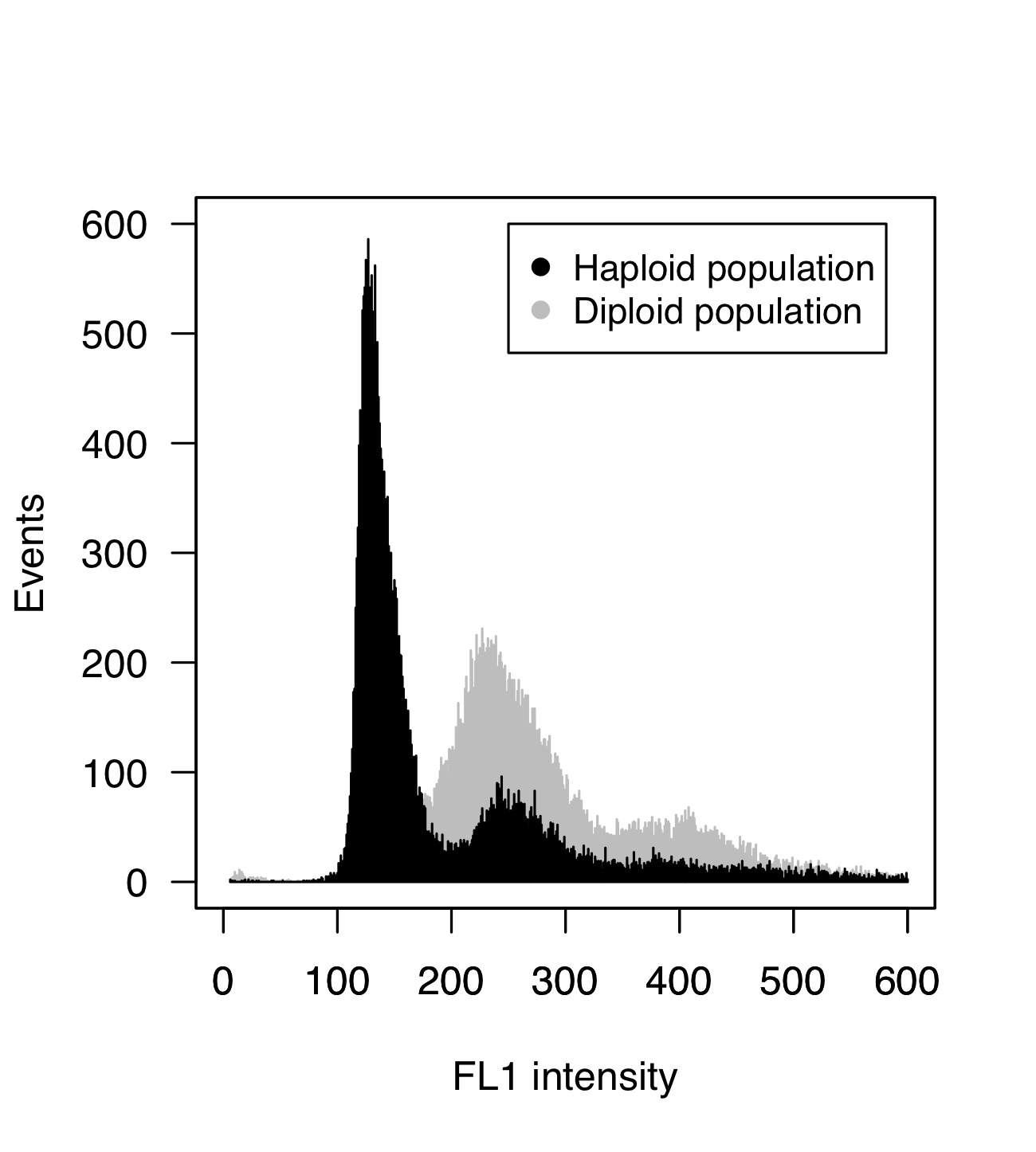

Supplement: Figure S3 — HU arrested haploid and diploid populations. Hydroxyurea is used to synchronize the cell cycle of populations. Presented are the measurement of 30 000 cells from a population composed entirely of haploids (black) and 30 000 cells from a population of diploids (grey). This method is not perfect, as some cells escape arrest. We have found the fraction of un-arrested cells to be fairly consistent, however, and as we focus our results on the difference between the ratio of haploids/diploids from one time point to another, this should not bias our conclusions. (TIFF) [file pone.0026599.s003.tif]

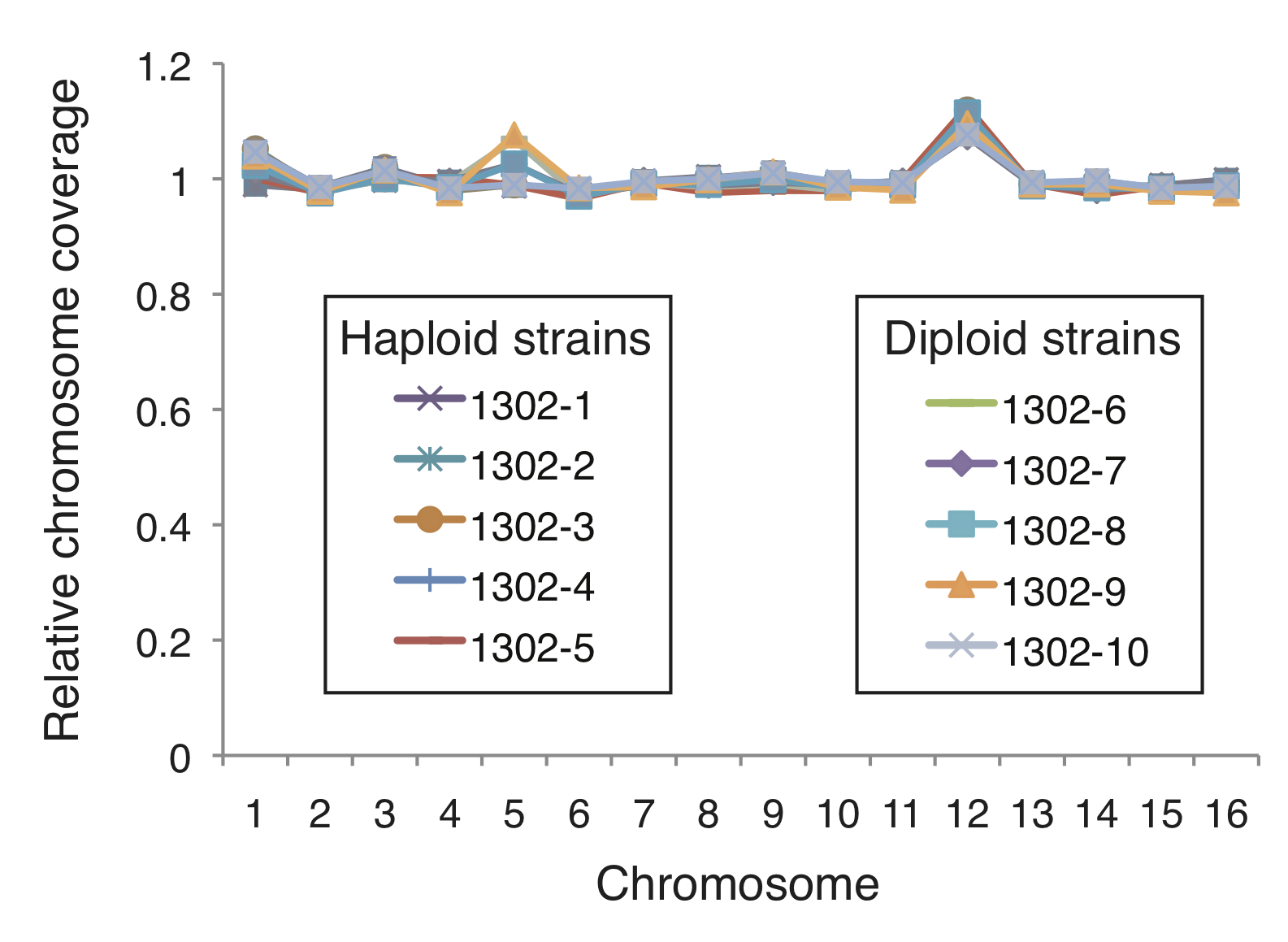

Supplement: Figure S4 — Relative chromosome coverage. From generation 1302, genomic DNA from five haploid and five diploid strains was extracted [1] and sequenced in 100 bp single-end fragments using Illumina's HighSeq 2000. Library preps followed standard Illumina protocols (2011 Illumina, Inc., all rights reserved), with each strain individually barcoded. The resulting genomic sequence data were processed using Illumina's CASAVA-1.8.0. Specifically, configureBclToFastq.pl was used to convert to fastq and separate the sequences by barcode (allowing one mismatched basepair). configureAlignment.pl was then used to align each sequence to the yeast reference genome (scergenome.fasta downloaded from the Saccharomyces Genome Database, http:). Finally configureBuild.pl was used to obtain coverage data. Average coverage per mapped site was 69.9 across the strains (with a minimum coverage per site of 16.3 for strain colony 4 from 1302 generations). Plotted for each strain is the proportion of sequenced sites from each chromosome relative to the proportion of known mapped sites on that chromosome within the reference genome. Although differences in ploidy cannot be detected with this method, whole-chromosome aneuploids would lead to larger shifts than observed (e.g., an additional chromosome should lead to 2x coverage in haploids and 1.5x coverage in diploids). We conclude that these strains are not aneuploids for whole chromosomes, including the chromosome IX aneuploidy that characterized their founding strain [2]. (The excess coverage on chromosome XII was also observed in an independent sequencing analysis of two strains from the knock-out deletion set, suggesting a common indel of regions on this chromosome or a mapping artefact.) (TIFF) [file pone.0026599.s004.tif]

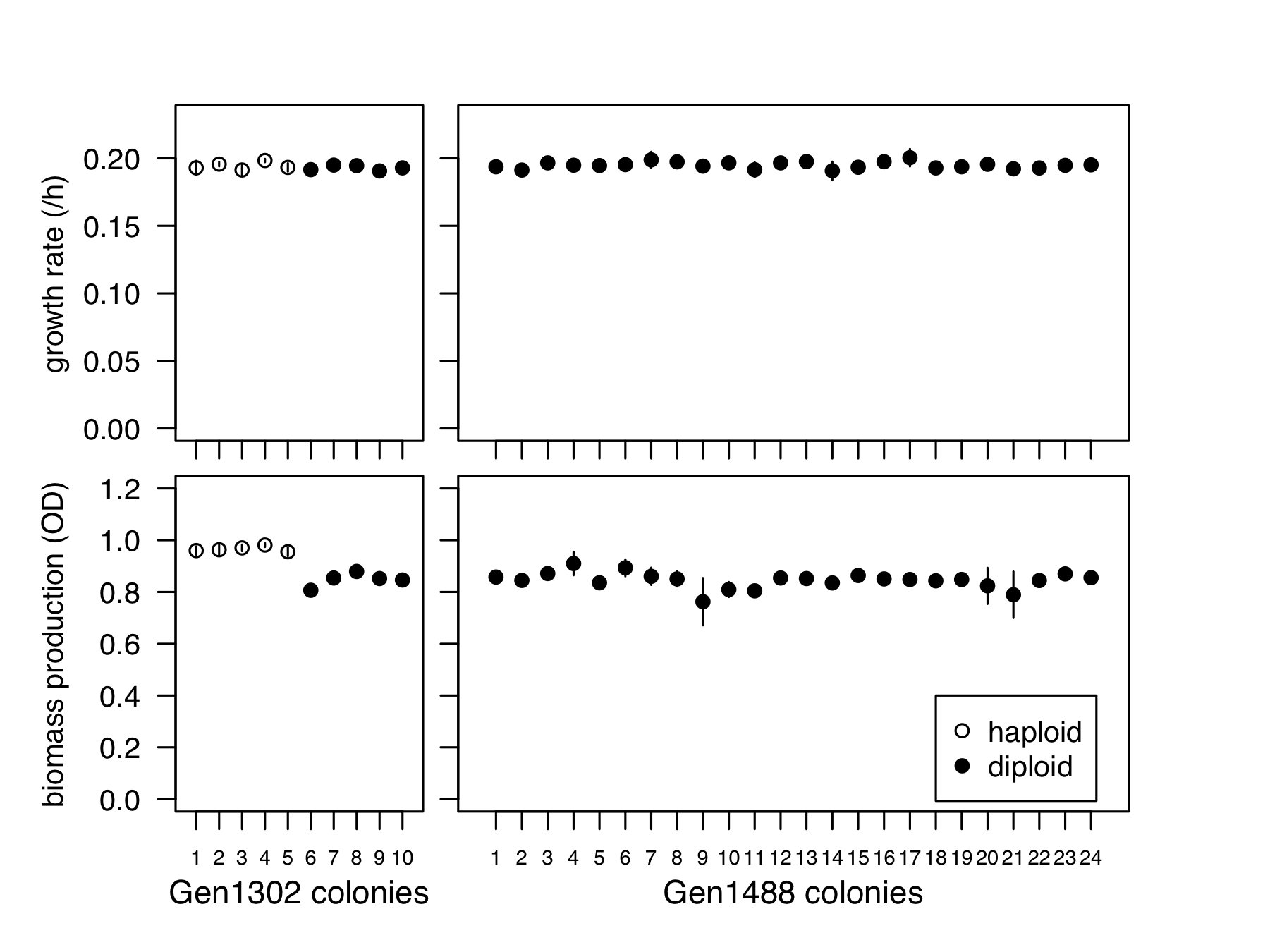

Supplement: Figure S5 — Fitness components of 1488 generation diploids do not predict diploid advantage. Growth rate and biomass production from 10 colonies (5 haploid and 5 diploid) isolated at 1302 generations and 24 diploid colonies isolated from 1488 generations were measured on a Bioscreen C Microbiology Workstation (Thermo Labsystems). Although only diploid colonies were present at 1550 generations, these fitness components do not predict a diploid advantage over the haploid colonies (first panel) that were present immediately before diploid takeover. (TIFF) [file pone.0026599.s005.tif]
