# Supplementary material for: Cryptic Fitness Advantage: Diploids Invade Haploid Populations Despite Lacking Any Apparent Advantage as Measured by Standard Fitness Assays
Source: PLoS One. 2011 Dec 9;6(12):e26599. doi: 10.1371/journal.pone.0026599 (PMC3235103; doi:10.1371/journal.pone.0026599)
Supplement: Table S1 — Cell volume correlates strongly with surface area, eccentricity and surface area:volume across colonies isolated across the time series. (PDF) [file pone.0026599.s006.pdf]

Table S2. Cell volume correlates strongly with surface area, eccentricity and surface area:volume across colonies isolated at generations 1023 & 1302

	r	statistic
volume & surface area	0.999	t_{21}=108.6, p<0.00001
volume & eccentricity	0.857	t_{21}=7.60, p<0.00001
volume & surface area:volume	-0.991	t_{21}=-34.5, p<0.00001
